# ACPA decreases non-small cell lung cancer line growth through Akt/PI3K and JNK pathways in vitro

**DOI:** 10.1038/s41419-020-03274-3

**Published:** 2021-01-11

**Authors:** Özge Boyacıoğlu, Elif Bilgiç, Cem Varan, Erem Bilensoy, Emirhan Nemutlu, Duygu Sevim, Çetin Kocaefe, Petek Korkusuz

**Affiliations:** 1 Hacettepe University, Graduate School of Science and Engineering, Department of Bioengineering, 06800 Beytepe Ankara, Turkey; 2 Hacettepe University, Faculty of Medicine, Department of Histology and Embryology, 06100 Sıhhiye Ankara, Turkey; 3 Hacettepe University, Faculty of Pharmacy, Department of Pharmaceutical Technology, 06100 Sıhhiye Ankara, Turkey; 4 Hacettepe University, Faculty of Pharmacy, Department of Analytical Chemistry, 06100 Sıhhiye Ankara, Turkey; 5 Hacettepe University, Faculty of Medicine, Department of Medical Biology, 06100 Sıhhiye Ankara, Turkey; 6 Present Address: Atılım University, Faculty of Medicine, Department of Medical Biochemistry, 06830 Gölbaşı Ankara, Turkey

**Keywords:** Cancer metabolism, Non-small-cell lung cancer, Apoptosis

## Abstract

Therapeutic agents used for non-small cell lung cancer (NSCLC) have limited curative efficacy and may trigger serious adverse effects. Cannabinoid ligands exert antiproliferative effect and induce apoptosis on numerous epithelial cancers. We confirmed that CB1 receptor (CB1R) is expressed in NSCLC cells in this study. Arachidonoylcyclopropylamide (ACPA) as a synthetic, CB1R-specific ligand decreased proliferation rate in NSCLC cells by WST-1 analysis and real-time proliferation assay (RTCA). The half-maximal inhibitory concentration (IC50) dose of ACPA was calculated as 1.39 × 10^−12^ M. CB1 antagonist AM281 inhibited the antiproliferative effect of ACPA. Flow cytometry and ultrastructural analyzes revealed significant early and late apoptosis with diminished cell viability. Nano-immunoassay and metabolomics data on activation status of CB1R-mediated pro-apoptotic pathways found that ACPA inhibited Akt/PI3K pathway, glycolysis, TCA cycle, amino acid biosynthesis, and urea cycle and activated JNK pathway. ACPA lost its chemical stability after 24 hours tested by liquid chromatography-mass spectrometry (LC–MS/MS) assay. A novel ACPA-PCL nanoparticle system was developed by nanoprecipitation method and characterized. Sustained release of ACPA-PCL nanoparticles also reduced proliferation of NSCLC cells. Our results demonstrated that low dose ACPA and ACPA-PCL nanoparticle system harbor opportunities to be developed as a novel therapy in NSCLC patients that require further in vivo studies beforehand to validate its anticancer effect.

## Introduction

Lung cancer associates with high mortality due to late diagnosis and rapid metastasis^1^. In total 80% of lung cancer cases are of non-small cell lung cancer (NSCLC) type^2^. Several therapeutic agents (Erlotinib, Gefitinib, Vandetanib, Dacomitinib, Icotinib, Afatinib, Bevacizumab, Crizotinib, and Ceritinib) targeting epidermal growth factor receptor (EGFR) or echinoderm microtubule-associated protein-like 4/anaplastic lymphoma kinase (EML4/ALK) were on clinical use for advanced or metastatic NSCLC^3–7^. However, systemic administration of these targeted therapies may cause serious side effects^8,9^ and effective treatment is not yet achieved^8^.

*Cannabis sativa* L. has more than 100 psychoactive components (terpenoids, flavonoids, fatty acids, etc.) known as cannabinoids^10,11^. Endocannabinoids are lipid structured endogenous cannabis ligands synthesized in mammalian peripheral tissues and generally act on classical cannabinoid receptors (CB1R and CB2R)^12,13^. The airway epithelium (bronchi and bronchioles)^14^, several immune system cells including lymphocytes, macrophages, and leukocytes^15,16^ contain endocannabinoid system. Endogenous and exogenous cannabinoids decrease proliferation on adenocarcinoma^17–21^ and squamous^20^ carcinoma subtypes of lung cancer. CB1R and CB2R levels in NSCLC tissues are higher than that of healthy ones^20^, whereas CB1R gene expression in bronchi is higher compared to CB2^22^. CB1R is expressed around 24% of NSCLC cases^17,23,24^. CB1R and CB2R mediate proapoptotic effect by inhibiting cAMP, activating ceramide synthase, and inhibiting protein kinase B (Akt) and phosphoinositide-3-kinase (PI3K) in breast^25^, gastric^26^ and prostate^27^ cancer cells. Arachidonoylcyclopropylamide (ACPA) is a synthetic CB1 agonist^28,29^ stimulating free oxygen radical-dependent autophagy by 5’-adenosine monophosphate-activated protein kinase (AMPK) activation in Panc1 pancreatic cancer cells^30^ and has antiproliferative effect on Panc1 cells^31–33^ while its impact on lung cancers remains unknown.

In this study we hypothesized that ACPA may exert a specific CB1R mediated reduction in proliferation and induction in apoptosis of NSCLC cells in vitro. If so, a novel biocompatible polymer-based nanoparticle system with low biodegradability for long-term controlled release of ACPA can be established for potential anticancer therapy. Primary objective of current study is to assess dose- and time-dependent antiproliferative and apoptotic effect and the mechanism of action of ACPA on CB1R expressing A549, H1299, H358, and H838 NSCLC cells by Water Soluble Tetrazolium-1 (WST-1), real time impedance-based proliferation (RTCA), flow cytometry (FCM), transmission electron microscopy (TEM), gas chromatography–mass spectrometry (GC/MS)-based metabolomics and Simple Western methods. Once the half-maximal inhibitory concentration (IC50) dose is set, the second objective is to design and optimize a novel biocompatible ACPA-loaded polycaprolactone (PCL) nanoparticulate delivery system to improve the stability and prolong the action of ACPA as a potential chemotherapeutic drug.

## Materials and methods

### Study design

A randomized in vitro observative study was designed including control-experiment groups as independent, proliferation-apoptosis measurements as dependent variables. Biological replicates were determined with power analysis (G-Power v3.1).

### Cell culture

A549 (CCL-185™)^18,34,35^ was cultivated with high glucose Dulbecco’s Modified Eagle (Gibco), H1299 (CRL-5803™)^34,36,37^, H358 (CRL-5807™)^35,36^, H838 (CRL-5844™)^37^, H1975 (CRL-5908™)^36,38^ and SW-1573 (CRL-2170™)^18,36^ were cultured in RPMI-1640 (Lonza Bioscience) (all provided from ATCC®). Culture conditions were kept at 37 °C under 5% CO_2_. In total 10% fetal bovine serum (Biological Industries), 2mM L-glutamine, 1% penicillin-streptomycin were utilized as supplements for both media.

### Quantitative real-time polymerase chain reaction (qRT-PCR)

CB1R and CB2R gene expression levels were documented in NSCLC lines^18,36^. Total RNA was isolated and cDNA synthesis was accomplished with QuantiTect^®^ Reverse Transcription Kit (Qiagen). qRT-PCR was done on a LightCycler^®^ 480 (Roche) instrument according to producers’ recommendations. Relative mRNA expression was assessed using PowerUp SYBR-Green Master Mix (Thermo Scientific) fluorescent dye. CB1R and CB2R levels were normalized to house-keeping gene (β-actin). Sequences of primers used are indicated in Supplementary Table 1.

### Immunocytochemistry

Indirect immune peroxidase labeling was carried out for CB1 (cat#C2866, Sigma-Aldrich) and CB2 (cat#HPA028718, Sigma-Aldrich) as previously done^33,39^. Percentage of labeled to total cell number on 25 areas at ×400 magnification was evaluated on automated microscope attached digital camera by image analysis program (Leica DMB6B, DFC7000T, LASV3 Wetzlar, Germany).

### Cell viability assays

Viability of 10^−^^6^-10^−12^ M ACPA (cat#1318, Tocris Bioscience)^30,31,33^ pre-treated NSCLC cells was determined by WST-1 (cat#11644807001, Roche). Controls were given media with 1% ethanol. Absorbance was measured on days 1, 2, and 3 using VersaMax Microplate Reader (Molecular Device) and examined with SoftMax Pro V5.2 Software at 450 and 630 nm wavelengths (*n* = 6).

IC50 of ACPA was assessed by xCELLigence (ACEA, Roche Applied System)^33^. High CB1R expressing cells were seeded in 96-well plates coated with gold microelectrodes recording impedance as “cell index”. After it exceeded 1.0, 10^−^^9^-10^−12^ M ACPA was applied to cells. 10^−^^5^-10^−7^ M CB1R antagonist AM281 (cat#1115, Tocris Bioscience) were added 15 min prior to ACPA for 3 days to evaluate CB1R agonism (*n* = 3).

### Apoptosis assays

NSCLC cells were exposed to IC50 dose of ACPA for 24, 36, 48, 72 hours and labeled with Annexin-V/Propidium Iodide (FITC Annexin Apoptosis Detection Kit I, cat#556547, BD Pharmingen) to determine the percentage of early, late apoptotic and necrotic cells by FCM (NovoCyte® 3000, ACEA Biosciences, Switzerland) (*n* = 4).

Cells kept for 24 hours with ACPA and normal medium were fixed in 2% glutaraldehyde and processed as previously performed^40^ for ultrastructural analysis by TEM (Jeol JEM-1400, Japan) and attached digital camera (Gatan, Germany).

### Simple Western

The total protein of A549 cells were harvested on following 24-hour incubation with ACPA (where maximum effect was observed) using RIPA buffer supplemented with phosphatase (cat#ab201113, Abcam) and protease (cat#78430, Thermo Scientific) inhibitors. Simple western assay was conducted following manufacturer’s guideline; 0.8 mg/ml (*n* = 4) of protein sample was separated on 12-230 kDa Wes Separation Module (cat#SM-W004, Protein Simple)^41^. Nano-immunoassay was performed using primary anti-human antibodies for Akt (S473, S474, and S472) (cat#mab2055), JNK (T183/Y185 and T221/Y223) (cat#mab1387) and phospho-specific Akt (cat#mab887) and phospho-specific rabbit anti-human JNK (cat#mab1205) (all from R&D Systems). Total and phospho protein expression was assessed for Akt and JNK (Wes, Protein Simple, USA).

### Intracellular metabolites analysis

Metabolomic profiling was conducted as reported previously^42,43^. Briefly, A549, H1299, H358, and H838 cells were incubated with ACPA and normal medium for 24 hours and extracted using methanol:water mixture (9:1, v/v) with internal standard (IS, 1 µg/ml myristic acid D27) and analyzed by GC-MS after methoxyamination and derivatization (*n* = 4).

### Analysis for stability of ACPA

ACPA was measured in all cells and cell supernatants on days 1, 2, and 3 by liquid chromatography-tandem mass spectrometry (LC-MS/MS) after its supplementation at 10^−6^ M (LCMS-8030, Shimadzu, Japan) (*n* = 3). Chromatographic separation was accomplished by using a C18 column (Hypersill-ODS4, 50 × 3.0 mm, 2.1 µm) with a mobile phase of acetonitrile and water (both containing 0.1% formic acid) with 0.3 ml/min flow rate. Daily calibration curve of ACPA was prepared at 6 different concentrations (1000–1.0 ng/ml) and constructed with the peak area ratio of ACPA to anandamide as IS versus concentration. Proteins in cell media with acetonitrile were centrifugated at 15000 rpm for 10 min and reconstituted using mobile phase including IS. Sample preparation for supernatant was done the same as in metabolomics except derivatization step.

Samples with ACPA at 10 µg/ml were prepared from stock ACPA solution, analyzed at 24 and 48 hours after storing at 37 °C and 4 °C (*n* = 3), and compared with freshly prepared ACPA solution at the same concentration.

### Preparation and characterization of ACPA-PCL nanoparticles

PCL MW:80,000 Da (cat#440744-250 G, Sigma-Aldrich) was used to prepare ACPA-loaded nanoparticles via nanoprecipitation method^44^. Acetonitrile:ethanol (9:1; v/v) including 0.1% (w/v) PCL and 0.01% (w/v) ACPA formed the organic phase was added to ultra-pure water (1:2 v/v) containing Pluronic F68 0.05% (w/v) dropwise at RT and subsequent colloidal mixture was magnetically stirred at 550 rpm for 30 min. Organic solvent was removed under vacuum to obtain final nanoparticle aqueous dispersion. in vitro characterization was performed by measuring particle size distribution, polydispersity index (PDI), zeta potential, ACPA encapsulation efficiency, and release as previously conducted by *Zeta Sizer Nano ZS* (United Kingdom)^45^ (*n* = 3). Encapsulation efficiency was determined after unbound ACPA was removed by centrifugation at 3500 rpm at RT. The supernatant was lyophilized and dissolved in dichloromethane which was then removed under nitrogen atmosphere to quantitatively analyze for ACPA content with LC-MS/MS. For in vitro release study, nanoparticle dispersion was kept in dialysis membrane immersed in release medium and stirred at 37 **°**C. Samples were analyzed with LC-MS/MS at 0, 1, and 4 hours and 1, 2, 3, 5, and 7 days. Release profile graph was obtained as %ACPA cumulative release.

### Cell viability assay for ACPA-PCL nanoparticles

Same protocol of RTCA was used. ACPA-PCL nanoparticles releasing low dose window were applied to cells once whereas ACPA was solely applied every day. Blank PCL nanoparticles were diluted to same volume as ACPA-PCL nanoparticles. Stable and prolonged effect of ACPA was observed (*n* = 3).

### Statistical analysis

Whole data exhibited normal distribution by Shapiro-Wilk test. One-way analysis of variance (ANOVA) and post-hoc Duncan’s test were used for comparison of parametric results. Student’s t-test was conducted for metabolomics and Simple Western. Descriptive data were presented as mean ± SEM. Spearman’s test was performed for correlation analysis. All data were evaluated within 95% confidence interval.

## Results

### NSCLC cells express high CB1R and low CB2R

**Fig. 1:**
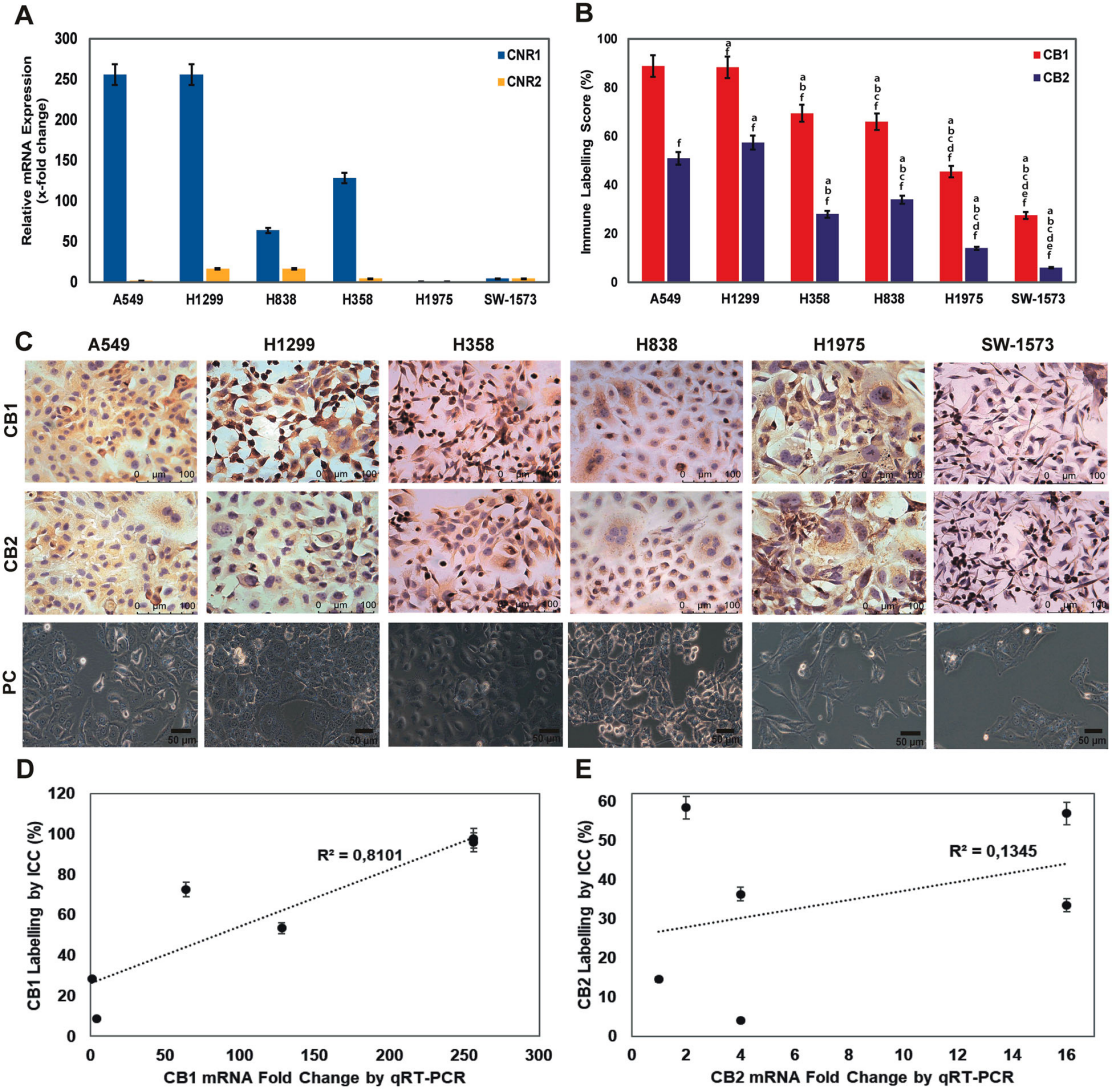
NSCLC cell lines express CB1R. **a** qRT-PCR analysis for relative mRNA fold change values of CB1R and CB2R genes relative to β-actin. The results are represented with 5% error. **b** Bar graph presenting the percentage of CB1 and CB2 indirect immune peroxidase-labeled cells with mean ± SEM, (*n* = 25), a, b, c, d, and e denote *p* < 0.05 comparing to A549, H1299, H358, H838, and H1975 cells respectively and f refers to *p* < 0.05 comparing CB2 to CB1 in each cell line, one-way analysis of variance (ANOVA). **c** Micrographs presenting CB1 and CB2 indirect immune peroxidase-labeled (scale bar, 100 µm) and phase-contrast (PC, scale bar, 50 µm) view of NSCLC A549, H1299, H358, H838, H1975, and SW-1573 cell lines, ×400. **d**, **e** Correlation graph of CB1 (**d**) and CB2 (**e**) receptor immune labeling and mRNA expression levels (correlation coefficient: R^2^, *p* = 0.024, and *p* = 0.824 respectively with Spearman’s test).

A549, H1299, H358 and H838 cells presented high CB1 and low CB2 mRNA expression and immune reactivity by qRT-PCR and immunocytochemistry respectively (Fig. 1a, b). No CB1 relative mRNA expression was noted for H1975 and SW-1573 cells (Fig. 1a). All cell lines presented significantly higher CB1 immune labeling when compared to CB2 (Fig. 1b) and exhibited mitotic figures, increased nucleus-to-cytoplasm ratio and cell pleomorphism under phase-contrast microscope (Fig. 1c). Labeling percentages presented strong positive correlation with CB1 mRNA expression levels (Fig. 1d) and a weak positive correlation with CB2 (Fig. 1e).

### IC50 dose of ACPA decreases proliferation and increases apoptosis on NSCLC cells via Akt and JNK pathways

**Fig. 2:**
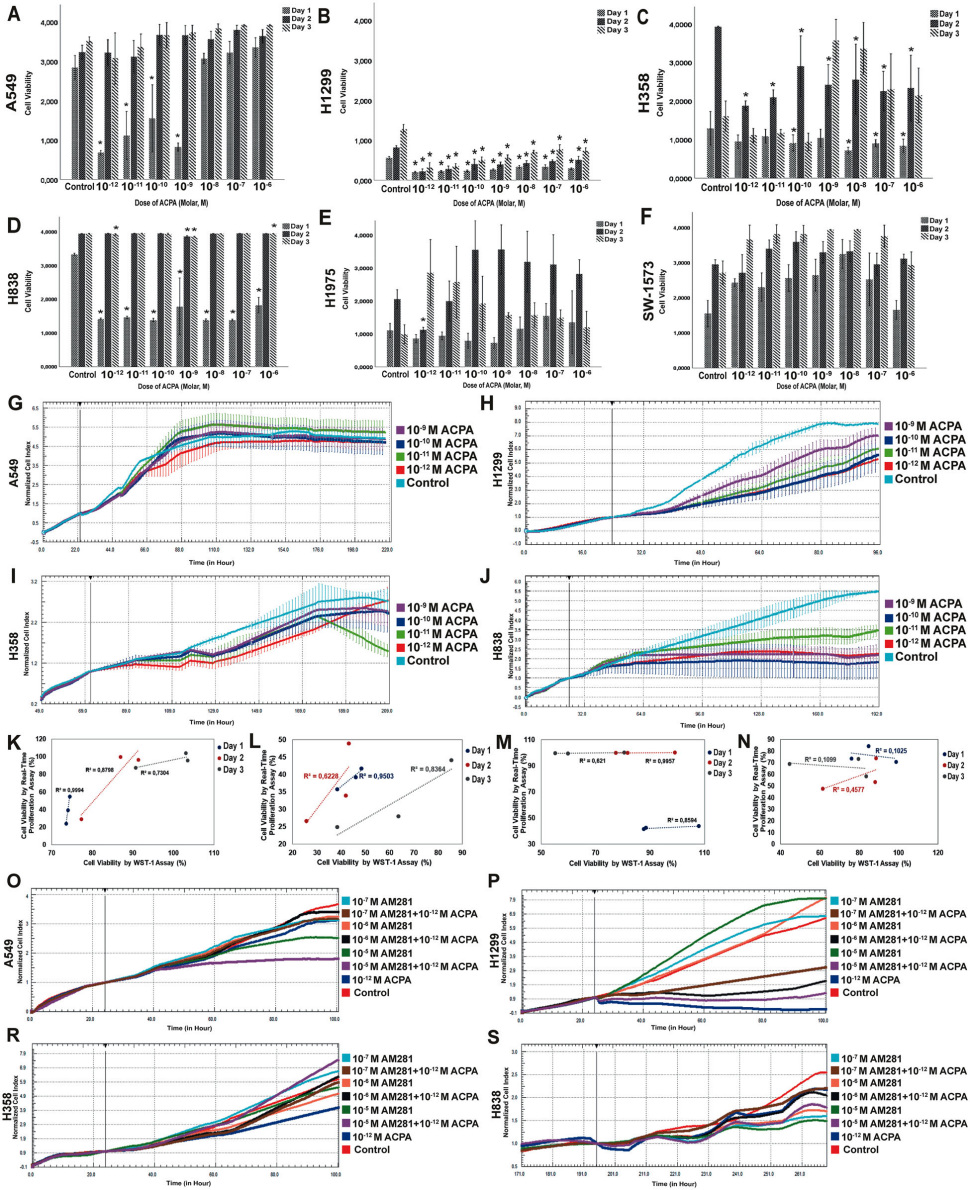
ACPA presents antiproliferative effect on NSCLC cells. Proliferation indices for (**a**) A549, (**b**) H1299, (**c**) H358, (**d**) H838, (**e**) H1975, and (**f**) SW-1573 cells by WST-1 presented with control and 10^−^^6^-10^−^^12^ M ACPA administered experiment groups from day 1 to 3 after treatment in mean ± SEM (*n* = 6, *p < 0.05 by one-way analysis of variance (ANOVA)). All proliferation data are shown in absorbance (A540 nm-A630 nm). Real-time proliferation indices of (**g**) A549, (**h**) H1299, (**i**) H358, and (**j**) H838 cells presented with control and 10^−^^9^-10^−^^12^ M ACPA administered experiment groups from 72 to 192 hours by xCELLigence (*n* = 3). Correlation graphs (**k**–**n**) present the relation between cell viability (%) by RTCA and WST-1 assay (correlation coefficient: *R*^2^, Spearman’s test). ACPA was applied at 10^−^^12^-10^−^^10^ M doses on (**k**) A549 (*p* **=** 0.015, *p* = 0.225 and *p* = 0.348 respectively), (**l**) H1299 (*p* **=** 0.143, *p* = 0.421 and *p* = 0.265 respectively), (**m**) H838 (*p* **=** 0.245, *p* = 0.042, and *p* = 0.422 respectively), and (**n**) H358 (*p* **=** 0.793, *p* = 0.527, and *p* = 0.785 respectively) cell lines. Real-time proliferation indices of (**o**) A549, (**p**) H1299, (**r**) H358, and (**s**) H838 cells with control, CB1 reverse agonist/antagonist AM281 and IC50 dose of ACPA administered groups for 74 hours by xCELLigence (*n* = 3).

ACPA presented maximum antiproliferative effect on high CB1R expressing A549 (Fig. 2a, g), H1299 (Fig. 2b, h), H358 (Fig. 2c, i), and H838 (Fig. 2d, j) cells at 10^−^^12^–10^−^^9^ M from day 1 to 3 by WST-1 and RTCA. Antiproliferative IC50 value of ACPA was calculated as 1.39 × 10^−^^12^ M for A549, H1299, and H358; it was calculated as 3.47 × 10^−11^ M in H838 cells. ACPA did not affect the proliferation of low CB1R expressing SW-1573 and H1975 cells (Fig. 2e, f). Antiproliferative effect of ACPA on A549, H1299, H358 and H838 lines did not significantly increase from day 1 to 3. A strong positive correlation was obtained between WST-1 and RTCA results in percent change in cell indices of A549 (Fig. 2k), H1299 (Fig. 2l), and H838 (Fig. 2m) on days 1, 2, and 3 following 10^−^^10^-10^−^^12^ M ACPA treatment. However, since the change of cell index pattern was not constant, a weak positive correlation between WST-1 and RTCA results was observed in H358 (Fig. 2n).

Co-application of 10^−^^6^-10^−7^ M AM281 with IC50 dose of ACPA inhibited the antiproliferative effect of ACPA and induced proliferation of A549 (Fig. 2o), H1299 (Fig. 2p), and H838 (Fig. 2s) cells on day 1. Co-application of 10^−^^6^-10^−7^ M AM281 with ACPA also increased H1299 and H838 cell proliferation from day 2 to 3. Co-application of AM281 with ACPA did not alter the proliferation of H358 cells (Fig. 2r).

**Fig. 3:**
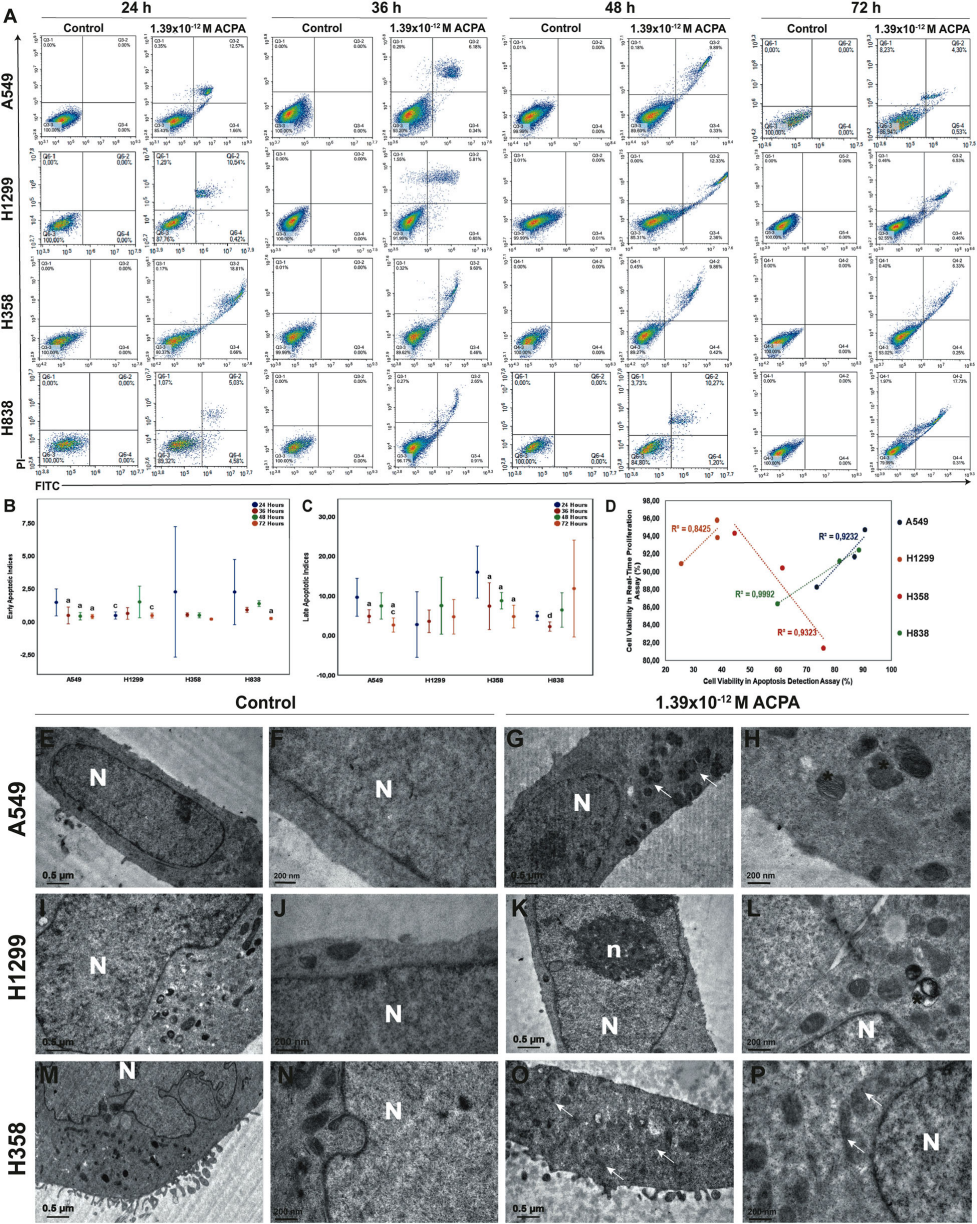
ACPA induces apoptosis on NSCLC cells. **a** Representative scatter dot-plot images of FCM depicting the effect of 1.39 × 10^−^^12^ M (IC50) dose of ACPA on NSCLC cells labeled with Annexin/PI. The percentage of viable, early, and late apoptotic to total ACPA-treated A549, H1299, H358, and H838 cells are shown with comparison to control at 24, 36, 48, and 72 hours. **b**, **c** Error bar graphs demonstrating early (**b**) and late (**c**) apoptotic indices for A549, H1299, H358, and H838 cells at 24, 36, 48, and 72 hours. **a**, **b**, **c**, and **d** demonstrate *p* < 0.05 comparing to 24, 36, 48, and 72 hours respectively, one-way analysis of variance (ANOVA). **d** Correlation graph between cell viability by RTCA and apoptosis by FCM following ACPA treatment of A549 (*p* = 0.01), H1299 (*p* = 0.667), H838 (*p* = 0.01), and H358 (*p* = 0.01) cells, Spearman’s test. **e**–**p** Left and right columns present the transmission electron micrographs of control and ACPA-treated groups respectively. All cancer cells exhibit cytoplasmic organelle composition of undifferentiated state with the lack of tubular systems. Note the presence of pleomorphic mitochondria with degenerated cristae and autophagic vacuoles in ACPA-treated groups (**g**, **h**, **k**, **l**, **o**, **p**). N: nucleus, n: nucleolus, (*): degenerated mitochondria, white arrow: autophagic vacuole. **e**, **g**, **i**, **k**, **m**, **o**, scale bar, 0.5 µm, **f**, **h**, **j**, **l**, **n**, **p**, scale bar, 200 nm.

Early (Fig. 3a, b, Supplementary Fig. 1) and late (Fig. 3a, c, Supplementary Fig. 1) apoptotic cell numbers by FCM were higher with IC50 dose of ACPA application at 24 to 72 hours in A549, H1299, H838, and H358 cells compared to untreated controls. Apoptotic effect of ACPA decreased significantly over time in A549 cells but remained constant from 24 to 72 hours in H1299, H358, and H838 cells (Fig. 3b).

A549 (Fig. 3e–h), H1299 (Fig. 3i–l) and H358 (Fig. 3m–p) cancer cells exhibited cytoplasmic organelle composition with obvious nuclear and nucleolar chromatin material and the lack of tubular systems by TEM. ACPA treated A549 (Fig. 3g, h), H1299 (Fig. 3k, l) and H358 (Fig. 3o, p) cells had giant mitochondria with degenerated cristae adjacent to varying amount of autophagic vacuoles. Ultrastructural morphology of ACPA-treated groups qualitatively supported the evidence of apoptosis by FCM. Cell viability percentages by RTCA presented positive correlation with total apoptosis rates by FCM in A549, H1299, and H838 cells and strong negative correlation in H358 cells (Fig. 3d).

**Fig. 4:**
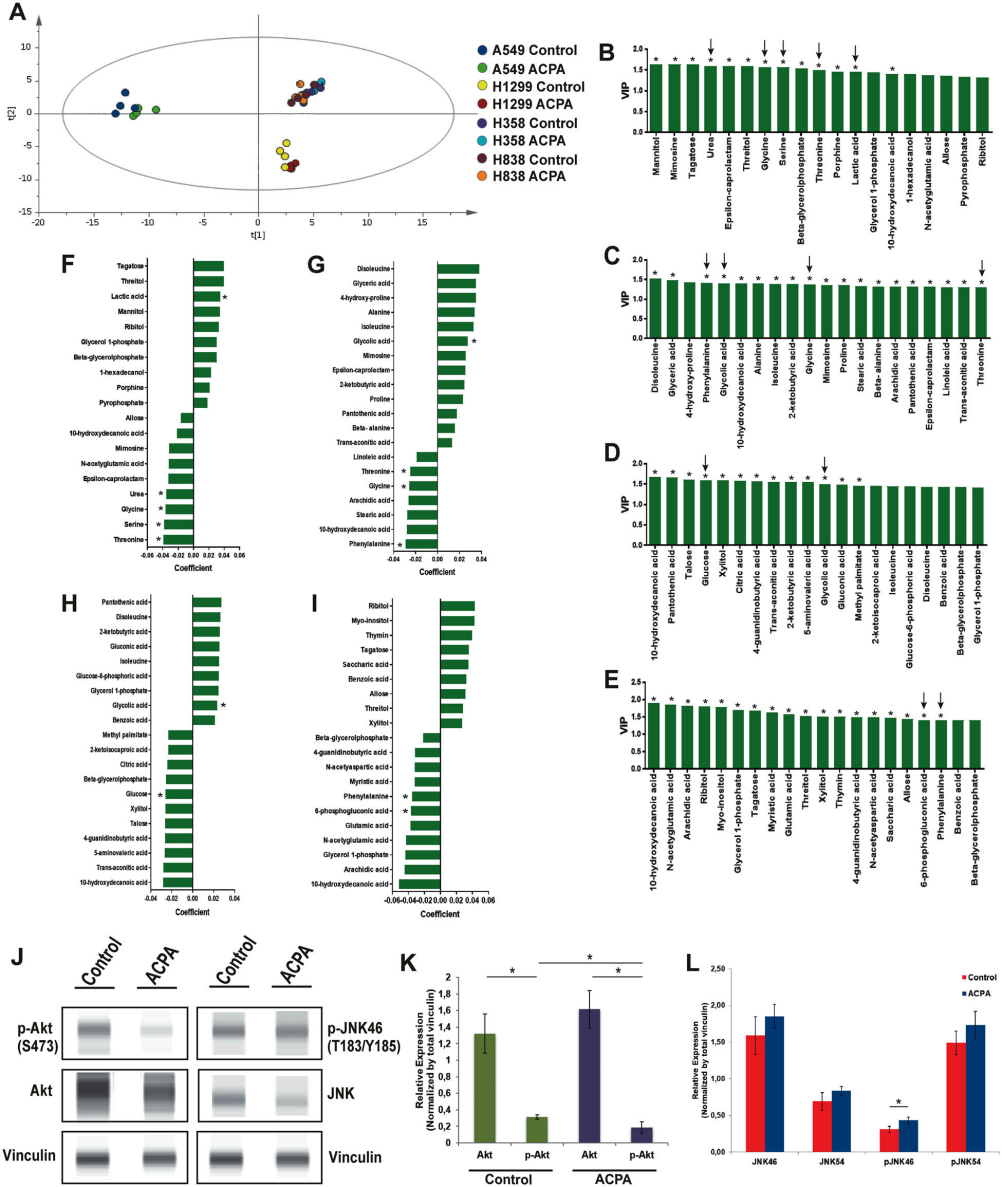
ACPA inhibits p-Akt, induces p-JNK and affects levels of specific metabolites in NSCLC lines. **a** Principal component analysis (PCA) score plot: Metabolomics profiling of control and ACPA-treated A549, H1299, H358, and H838 cells. **b** Changes in variable importance in projection (VIP) values for 19 metabolites in A549 cells. **c**, **d**, **e** Changes in VIP values for 20 metabolites in H1299, H358, and H838 cells. Significantly changed metabolites (*p < 0.05, indicated by arrows) were matched to apoptotic pathways. **f**, **g**, **h**, **i** Increase and decrease in several metabolites of ACPA-treated A549, H1299, H358, and H838 cells (*p < 0.05). **j** Simple Western showing total Akt, p-Akt (S473), total JNK46 and JNK54 and p-JNK46 and p-JNK54 (T183/Y185) in A549 cells at 24 hours after treatment with IC50 dose of ACPA. **k** Relative expression levels of Akt and p-Akt for control and ACPA-treated A549 cells after normalization by total vinculin protein. **l** Relative expression levels of JNK (46 and 54 kDa) and p-JNK for control and ACPA-treated A549 cells after normalization by total vinculin protein. *p < 0.05, Student’s t-test. All tests were done in quadruplicates.

**Fig. 5:**
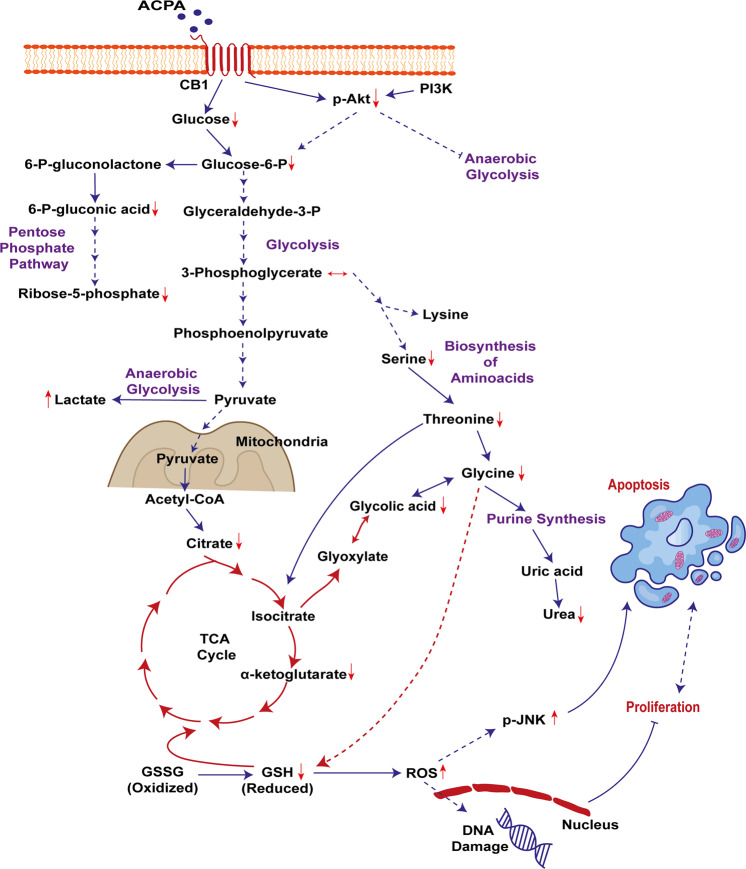
ACPA promotes Akt and JNK signaling pathways and affects several cellular processes in NSCLC lines. Critical metabolites in glycolysis, pentose phosphate, amino acid biosynthesis, and TCA cycle affected in A549, H1299, H358, and H838 cells on day 1 after IC50 dose of ACPA treatment.

Metabolites of ACPA-treated groups related to glycolysis, amino acid biosynthesis, TCA, and/or urea cycles altered at different levels (Fig. 4a–e, Supplementary Fig. 2). Glucose-6-phosphate (glycolysis), 6-phospho-gluconic acid, ribose-5-phosphate (pentose phosphate pathway), citrate, α-ketoglutarate (TCA cycle), serine, threonine, glycine (amino acid biosynthesis), urea, glycolic acid (glyoxylate pathway) and reduced glutathione levels decreased (Fig. 4b, f) in NSCLC cells which are also in association with significant p-Akt degradation (Fig. 4j, k) and p-JNK46 activation (Fig. 4j, l) promoting apoptosis in ACPA-treated A549 cells comparing to untreated control on day 1 by Simple Western analysis. ACPA increased lactic acid levels comparing to untreated control in A549 cells. ACPA significantly decreased phenylalanine, glycine, glucose, α-ketoglutarate, and 6-phospho-gluconic acid affecting the glycolytic and amino acid flux in H1299 (Fig. 4c, g), H358 (Fig. 4d, h), and H838 (Fig. 4e, i) cells comparing to untreated control. ACPA reduces proliferation and induces apoptosis via Akt/PI3K and JNK pathways, glycolysis, TCA cycle, amino acid biosynthesis and urea cycle (Fig. 5).

### ACPA became unstable after 24 hours

**Fig. 6:**
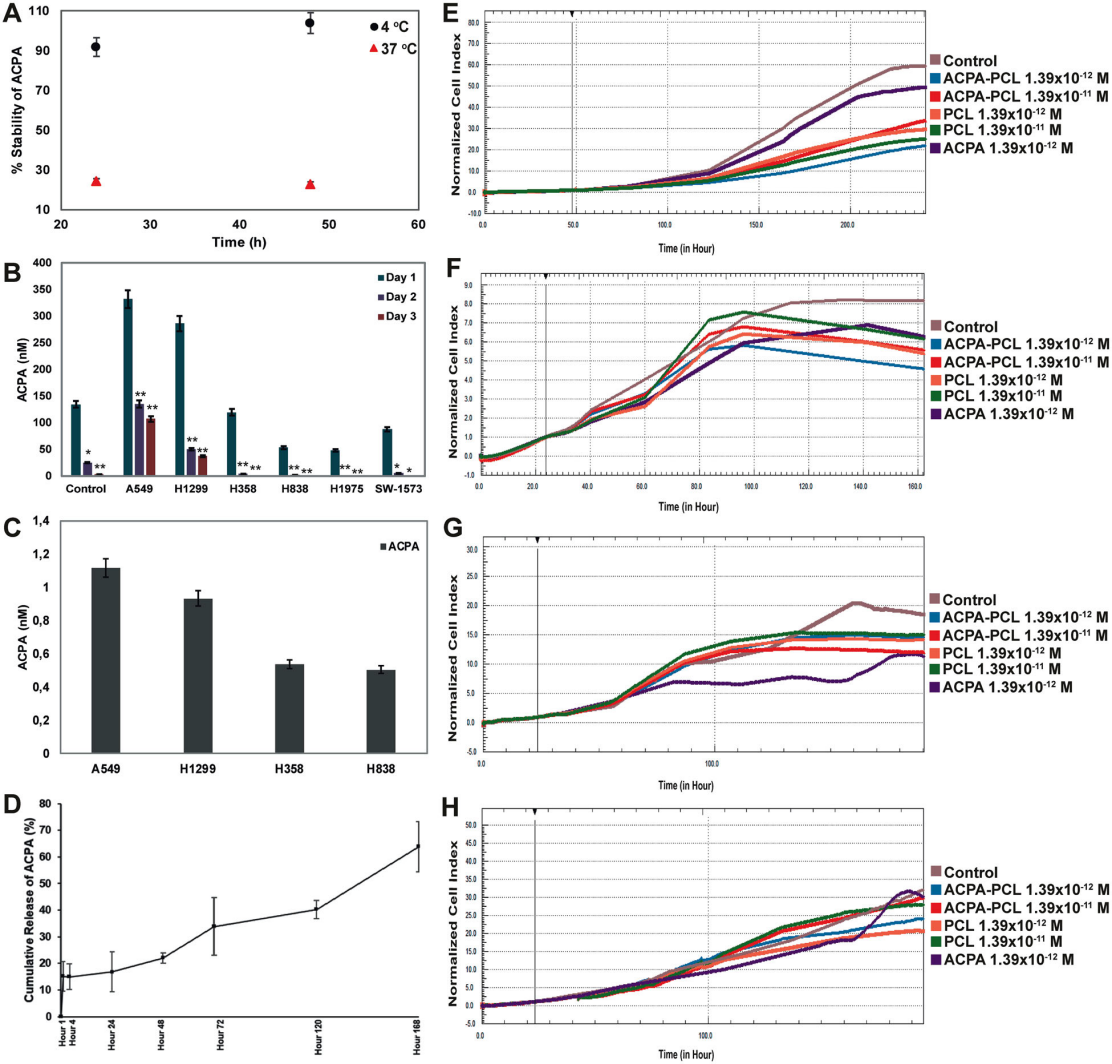
ACPA is unstable after 24 hours and ACPA-PCL nanoparticle system provides a sustained release. **a** Stability of ACPA in percentage under 4 °C and 37 °C on days 1 and 2. **b** Amount of ACPA (nM) in cell-free and cell-containing media in control and A549, H1299, H358, H838, H1975, and SW-1573 cells on days 1, 2 and 3, *p < 0.01, **p < 0.001 by one-way analysis of variance (ANOVA). Results are represented with mean ± SEM. **c** Amount of intracellular ACPA (nM) in A549, H1299, H358, and H838 cell lines on day 1 after administration. Values were normalized to normal culture media. **d** Cumulative release of ACPA from PCL nanoparticles (%) at hours 1, and 4, and days 1, 2, 3, 5, and 7. **e**, **f**, **g**, **h** Normalized cell indices over time assessed with RTCA for (**e**) A549, (**f**) H1299, (**g**) H358, and (**h**) H838 cells. Untreated controls, ACPA-PCL nanoparticles releasing low dose window, diluted empty PCL nanoparticles and 1.39 × 10^−^^12^ M dose of ACPA-treated groups are shown for each cell line. All experiments were conducted in triplicates.

ACPA lost its chemical stability at 37 °C when compared to 4 °C on day 1 and 2 (Fig. 6a). Amount of ACPA in cell-free and cell-containing culture media decreased from day 1 to 3 (Fig. 6b). Intracellular amount of ACPA was measured between 0.4 and 1.2 nM on day 1 (Fig. 6c).

### ACPA-PCL nanoparticles were characterized

Mean particle size, PDI, and zeta potential of APCA-PCL nanoparticles were revealed as 162.2 ± 2.3 nm, 0.251 ± 0.008, and −29.4 ± 0.6 mV, respectively. A total of 39.9 ± 14.7% ACPA was loaded into the PCL nanoparticles and its sustained cumulative release from the nanoparticles has been noted as 63.9% for a period of 7 days (Fig. 6d).

### ACPA-PCL nanoparticles provided long-term antiproliferative effect on NSCLC cells

ACPA-PCL nanoparticle-treated groups significantly reduced proliferation of A549 cells from day 2 to 8 and H1299 cells from day 1 to 7 (Fig. 6e, f). ACPA-PCL nanoparticles did not have a statistically significant antiproliferative effect on H358 and H838 cells until day 5. Control groups were found significantly higher than ACPA-PCL nanoparticle-treated H358 and H838 cells from day 5 to 7 (Fig. 6g, h).

## Discussion

In this study A549, H1299, H358, and H838 NSCLC cells presented high CB1R mRNA expression and exhibited diffuse CB1 immune labeling that quantitatively correlated with qRT-PCR results. Our data is in line with previous studies investigating CB1 mRNA expression in A549^18,34,35^, H1299^34,36,37^, H358^35,36^, and H838^36,37^ cells also supporting that CB1Rs are expressed higher than CB2 in bronchi in lungs^22^. Cannabinoid trafficking occurs within a dynamic milieu and cannabinoid receptors cycle constitutively between plasma membrane and cytoplasm^46–48^ which may also reflect lower CB2 immune labeling in NSCLC cells.

Here we report that 10^−9^–10^−^^12^ M doses of ACPA significantly reduced proliferation of A549, H1299, H358, and H838 cells in a dose- and time-dependent manner. Antiproliferative IC50 dose of ACPA was determined as 1.39×10^−^^12^ M by RTCA which also exerted antiproliferative effect by decreasing viability, increasing early and late apoptosis mainly on day 1 by FCM. Specific CB1 antagonist AM281 reversed the antiproliferative effect of ACPA on A549, H1299, and H838 cells on day 1. Antiproliferation and apoptosis data significantly correlated with CB1R mRNA expression. Previous reports^49,50^ regarding the impact of variant CB1 antagonists are coherent with our findings. Our research group previously determined IC50 dose of ACPA as 9.3 × 10^−6^ M on CB1R expressing Ishikawa endometrial cancer cells at 46 hours in vitro which induced 8.9% early and 62.8% late apoptosis by FCM^33^. In total 10^−^^4^–4.0 × 10^−4^ M ACPA decreased proliferation^32^ and triggered autophagy in Panc1 cancer cells^30^. Combined treatment of 9.0 × 10^−5^ M ACPA with 2.0 × 10^−7^ M gemcitabine reduced proliferation in Panc1 cells by crystal violet staining^31^. Herein, ACPA provided an efficient antiproliferation on 4 different CB1R expressing NSCLC cells at a lower dose range comparing to previous endometrial and pancreatic cancer cells. Furthermore, we determined a low IC50 dose of ACPA for targeting NSCLC in vitro for the first time. So far, in vitro studies have been conducted to elucidate antiproliferative and/or apoptotic effect of cannabinoids on A549^18,24,34,51–53^, H358^51^, H1299^37^, and H838^37^ cells. Here, 10^−8^–10^−6^ M ACPA did not affect NSCLC cell viability. High doses of cannabinoids may decrease cellular response by inducing receptor internalization/desensitization^54^ and exhibit adverse effects mainly in vitro studies^55^ including morphological changes in cell lines and inhibition of drug metabolism^55,56^. Taken together, ACPA may act as an efficient synthetic anticancer drug candidate functioning purely via CB1R to induce apoptosis in NSCLC cells at a low dose window comparing to other cannabis ligands.

ACPA induces cell death on NSCLC cells via Akt/PI3K, glycolysis, pentose phosphate pathways; amino acid biosynthesis, urea, and TCA cycles in our study. Degradation in various metabolites can be associated with p-Akt degradation and p-JNK activation in ACPA-treated A549 cells comparing to control on day 1 by Simple Western. Exogenous cannabinoids have been reported to mediate antitumor activity via Akt/PI3K inhibition^13,17,18,34,57–61^ and JNK activation^18,27,61–63^ through CB1R and/or CB2R in various cancer cells including NSCLC. ACPA induced autophagy by activating AMPK, inhibiting glycolysis^30^ and proliferation in Panc1 cells by blocking pyruvate kinase-2 which is found in lung and pancreatic islets^32^. Our data regarding p-Akt degradation and p-JNK activation is coherent with previous findings reporting antiproliferation nature of Akt and JNK function. Lung tumors can be characterized with glycine/serine/threonine upregulation^64,65^, pentose phosphate and glycolysis pathways, and TCA cycle^66^ regulating cell proliferation and reduced glutathione as an oxidative stress marker. Those findings could improve our understanding of metabolomic results that ACPA can stimulate apoptotic pathways by reducing critical metabolites in NSCLC cells.

ACPA can penetrate the cell membrane to bind its receptor in any intracellular location and intracellular/extracellular ACPA levels matter for its antiproliferative effect. Therefore, we initially demonstrated that amount of ACPA in-cell free and cell-containing culture media diminished after 24 hours at 37 °C which accounts for the instability of ACPA upon storage. We prepared ACPA-PCL nanoparticles according to IC50 dose of ACPA to mimic the time-dependent release within the cellular milieu and noted approximate ACPA loading efficiency as 40% with a cumulative release of 64% for 7 day-period. Cannabinoids are lipophilic molecules with low solubility^67^ and drug delivery systems have recently been developed with cannabinoids due to shorter half-lives^11,68–70^. Various cannabinoids were loaded with 60–100% efficiency into lipid^71,72^ and polymer-based^69,73–76^ nanoparticles showing about 60–100% cumulative release after 7 hours-30 days of loading. PCL is a biopolymer approved by the FDA for therapeutic use in drug delivery systems due to its biocompatibility and hydrophobicity facilitating encapsulation of poorly soluble molecules such as ACPA^77^. Although our loading efficiency data was lower than expected, the releasing profile of ACPA was evaluated within the range stated in the literature which thus provided a basis for our novel ACPA-PCL nanoparticle system. As ACPA has poor stability in handling and storage conditions, PCL provided an optimum matrix structure in which ACPA could be encapsulated based on weak electrostatic interactions, mostly within the polymer matrix but also adsorbed on the nanoparticle surface. As PCL nanoparticles do not have a lipid core, loading efficiency was lower than lipidic nanocarriers^71,72^. However, PCL compensates the lower loading data and suggests a promising nanomedicine to develop ACPA as a therapeutic product. Moreover, hydrophobicity is the main parameter controlling the release rate of ACPA from PCL nanoparticles which was observed as a slow-release up to 7 days. Complete release is not yet achieved in this 7-day period suggesting a longer release time. Initial release may be a result of the burst effect of ACPA adsorbed on nanoparticle surface followed by gradual release of encapsulated ACPA governed by diffusion, partition coefficient of ACPA, and matrix erosion of the nanoparticle liberating the encapsulated drug.

PCL nanoparticles releasing ACPA also reduced proliferation of all cells at different time intervals. PCL nanoparticles releasing exogenous cannabinoids inhibited proliferation of basophilic leukemia^76^, colon^76,78^, breast^69,79^, and NSCLC^78^ cancer cells. Here we provided ACPA-PCL nanoparticles releasing low dose window inducing a significant antiproliferation on NSCLC cells in vitro.

Low doses of ACPA and ACPA-PCL nanoparticles have antiproliferative and/or apoptotic effects on NSCLC cells via Akt/PI3K and JNK pathways. Since this is a preliminary study, results were limited to in vitro conditions which requires to be completed with further functional in vitro and in vivo studies. This limitation, however, does not obstruct further in vivo and clinical researches since statistical accuracy and parametric distribution were validated. Moreover, RTCA has been reported as one of the most reliable in vitro methods permitting the assessment of potential personalized therapeutics with high sensitivity and specificity before clinic^80^. Lack of evaluation of CB1R and CB2R expressions in primary lung epithelium in our study may be a limitation. Still, CB1R mRNA expression results contribute to the literature. Lack of assessing p-Akt/p-JNK expressions in ACPA-treated A549 cells in the presence of Akt/JNK inhibitors is a limitation which needs to be analyzed in further studies. Since ACPA has a lipophilic characteristic and a short half-life, maintaining its stability was difficult throughout in vitro studies. To overcome this limitation, we first developed ACPA-PCL nanoparticles. In conclusion, anticancer effect of ACPA and novel ACPA-PCL nanoparticle system through CB1R agonism could represent a promising nanomedicine candidate for in vivo studies and further clinical trials for the eventual reduction of potential adverse effects of systemic EGFR or EML4/ALK targeting chemotherapeutics that are used for lung cancer in the clinic.

## Supplementary information

Supplementary Figure Legends

Supplementary Table 1

Supplementary Figure 1

Supplementary Figure 2
