# Supplementary material for: ACPA decreases non-small cell lung cancer line growth through Akt/PI3K and JNK pathways in vitro
Source: Cell Death Dis. 2021 Jan 11;12(1):56. doi: 10.1038/s41419-020-03274-3 (PMC7801394; doi:10.1038/s41419-020-03274-3)
Supplement: Supplementary file 1 — Supplementary Figure Legends [file 41419_2020_3274_MOESM1_ESM.docx]

**Supplementary Figure Legends**

**Supplementary Table 1.** Primer sequences designed for qRT-PCR.

**Supplementary Fig. 1.** Raw data of scatter dot-plot images of FCM representing the effect of IC50 dose of ACPA on NSCLC cells labelled with Annexin/PI.

**Supplementary Fig. 2. a, b, c, d** Raw data of principal component analysis (PCA) score plot: Metabolomics profiling of control and ACPA-treated **(a)** A549, **(b)** H1299, **(c)** H358 and **(d)** H838 cells. **e, f, g, h** Raw data of changes in variable importance in projection (VIP) values for **(e)** A549, **(f)** H1299, **(g)** H358 and **(h)** H838 cells.
